# Live‐cell super‐resolution imaging of actin using LifeAct‐14 with a PAINT‐based approach

**DOI:** 10.1002/pro.4558

**Published:** 2023-02-01

**Authors:** Haresh Bhaskar, Dirk‐Jan Kleinjan, Curran Oi, Zoe Gidden, Susan J. Rosser, Mathew H. Horrocks, Lynne Regan

**Affiliations:** ^1^ The School of Biological Sciences University of Edinburgh Edinburgh UK; ^2^ EaStCHEM School of Chemistry University of Edinburgh Edinburgh UK; ^3^ Centre for Synthetic and Systems Biology and UK Centre for Mammalian Synthetic Biology, School of Biological Sciences University of Edinburgh Edinburgh UK; ^4^ Department of Genome Sciences University of Washington Seattle Washington USA

**Keywords:** actin, LifeAct, live‐cell imaging, single‐molecule localization microscopy (SMLM), super‐resolution (SR) imaging

## Abstract

We present direct‐LIVE‐PAINT, an easy‐to‐implement approach for the nanoscopic imaging of protein structures in live cells using labeled binding peptides. We demonstrate the feasibility of direct‐LIVE‐PAINT with an actin‐binding peptide fused to EGFP, the location of which can be accurately determined as it transiently binds to actin filaments. We show that direct‐LIVE‐PAINT can be used to image actin structures below the diffraction‐limit of light and have used it to observe the dynamic nature of actin in live cells. We envisage a similar approach could be applied to imaging other proteins within live mammalian cells.

## 1 INTRODUCTION

Molecules can be visualized at a resolution below the diffraction‐limit of light (~200 nm) using a variety of optical techniques (Hell & Wichmann, 1994; Betzig et al., 2006; Rust et al., 2006) that are collectively grouped under the term super‐resolution (SR) microscopy. In single‐molecule localization microscopy (SMLM) approaches, the spatiotemporal separation of individual emitters is achieved via the stochastic activation of fluorophores, each of which can be localized with nanometer accuracy (for a comprehensive review of techniques, see [Horrocks et al., 2014]). In point accumulation for imaging in nanoscale topography (PAINT), fluorescent molecules temporarily bind to surfaces, such as membranes, and are localized allowing for SR imaging (Sharonov & Hochstrasser, 2006). Since the initial demonstration of this strategy, variations on the method have been developed by several different groups (Giannone et al., 2013; Whiten et al., 2018; Sanders et al., 2022). DNA‐PAINT has proven particularly useful and has been applied to imaging DNA origami arrays *in vitro*, and proteins in fixed samples (Schnitzbauer et al., 2017; Guo et al., 2019). As DNA‐PAINT relies on tagging biomolecules with unique oligonucleotide sequences, it can also be used for multiplexed imaging (Jungmann et al., 2014). An analogous approach, peptide‐PAINT, has also been developed using peptide–peptide interaction pairs (Eklund et al., 2020).

The requirement for fixation and permeabilization, however, generally precludes the use of either DNA‐PAINT or peptide‐PAINT for live‐cell imaging, although in some cases DNA‐PAINT has been used to image membrane proteins on the outer surface of live cells (Strauss et al., 2018). To circumvent the limitation of cell fixation and permeabilization, we recently developed Live cell Imaging using reVersible intEractions‐PAINT (LIVE‐PAINT), a method that uses transient protein–protein interactions to perform SR imaging in live cells (Oi et al., 2020a). In this approach, the protein‐of‐interest is genetically fused to a short peptide sequence, and an SR image is generated as this is transiently bound by an interacting peptide fused to a fluorescent protein (Oi et al., 2020b). While useful for imaging targets in live cells, the LIVE‐PAINT technique has only been demonstrated in live yeast, and also requires the modification of the target protein, which may perturb its structure and function. To circumvent this limitation, we introduce direct‐LIVE‐PAINT. Rather than relying on tagging the protein‐of‐interest with a peptide sequence, direct‐LIVE‐PAINT uses genetically‐encoded fluorescent peptides that directly interact with the unmodified, endogenous protein, enabling SR imaging in live mammalian cells.

**FIGURE 1:**
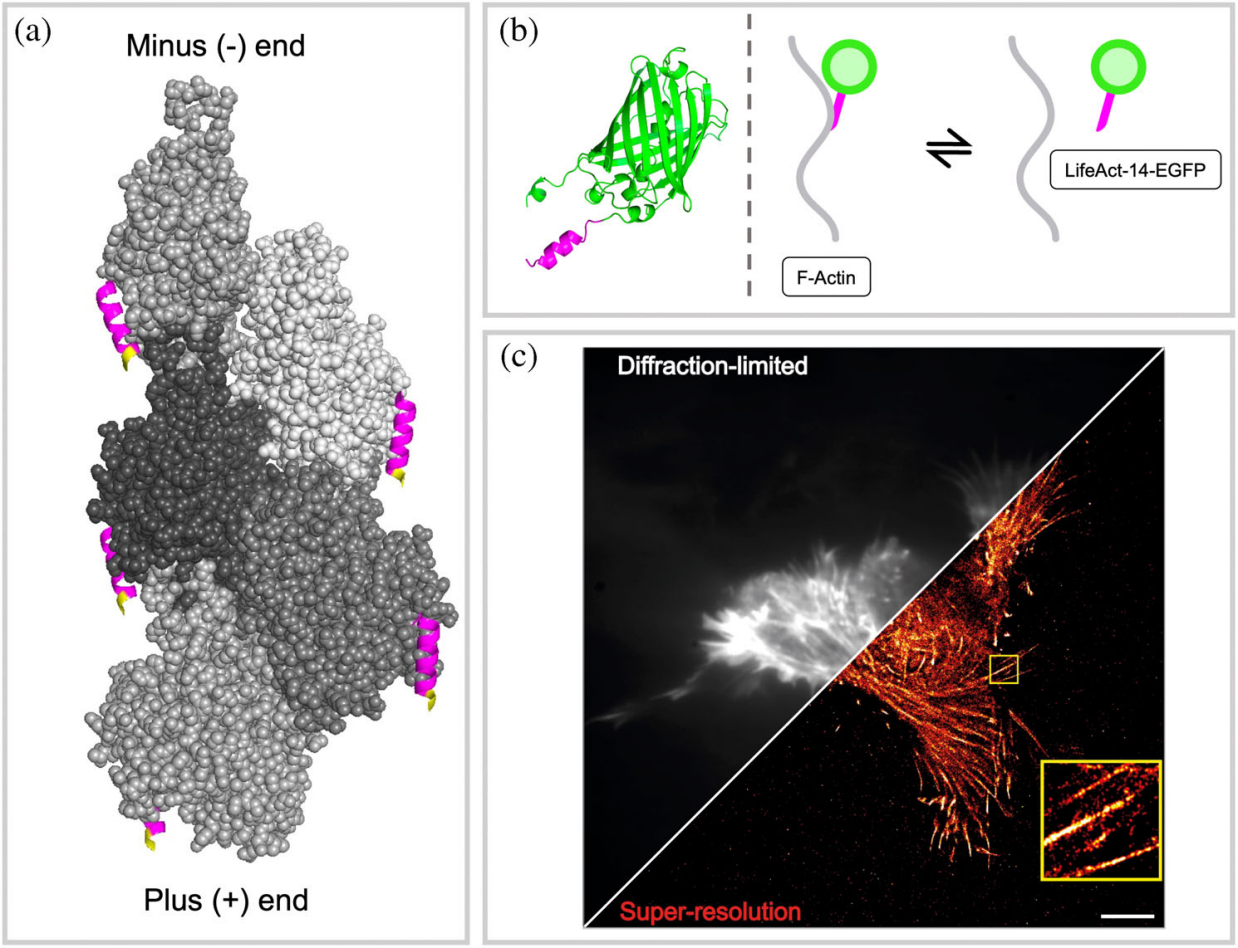
(a) Cryo‐EM structure of LifeAct (17 AA) bound to F‐actin. Five monomers of actin are shown in space‐filling representation in shades of gray. Each actin monomer is shown bound to the LifeAct peptide, which is shown in magenta in the ribbon representation. The three residues at the C‐terminus, colored yellow, indicate the amino acids that are missing from LifeAct‐14. Structure 7AD9 was retrieved from the PDB (Belyy et al., 2020). (b) Cartoons showing a predicted structure of the LifeAct‐14‐EGFP construct (LifeAct‐14, magenta; EGFP, green) (Jumper et al., 2021) and illustrating the reversible nature of the binding between LifeAct‐14‐EGFP and actin filaments. EGFP sequence retrieved from FPbase (ID: R9NL8) (Lambert, 2019). Structures visualized using PyMOL (the PyMOL molecular graphics system, version 2.0 Schrödinger, LLC.). (c) A HEK293 cell, transiently transfected with a plasmid expressing LifeAct‐14‐EGFP. The distinctive actin filament structure is clearly visible in the region of interest (ROI, yellow box). The diagonal line separates diffraction‐limited and super‐resolution (SR) imaging. SR image colored “red hot.” Diffraction‐limited image was obtained by Z‐projecting the time‐course images (100 s, 50 ms exposure). 5 𝜇m scale bar

Actin is an abundant protein that is a component of the eukaryotic cytoskeleton. Its dynamic conversion between the monomeric (G‐actin) and the filamentous (F‐actin) state underlies key cellular functions. New ways to image F‐actin in live cells are especially useful since when directly fused to a fluorescent protein, actin is not fully functional. One probe, termed LifeAct, uses the N‐terminal 17 amino acids of an actin‐binding protein Abp140 fused to EGFP to label actin (Riedl et al., 2008). Although widely used to label actin in live and fixed cells (Han et al., 2019; von Chamier et al., 2021), artifacts have been noted with LifeAct (Kiuchi et al., 2015; Flores et al., 2019). Most notably, it has been reported that LifeAct exhibits a higher affinity for G‐actin than for F‐actin and that this differential affinity perturbs the equilibrium, causing artifacts (Courtemanche et al., 2016; Flores et al., 2019). Kumari et al. recently reported that a peptide lacking the C‐terminal 3 amino acids of LifeAct, which they named LifeAct‐14, binds to F‐actin with similar affinity (*K*_*D*_ = 1.2 μM) to LifeAct, and does not exhibit preferential binding to G‐actin (Kumari et al., 2020). They, therefore, reasoned it would be less perturbing to cellular function (actin assembly) than LifeAct itself (Figure 1a).

Based on its low micromolar affinity and negligible perturbative effects, we postulated that expression of LifeAct‐14‐EGFP fusion could be used to obtain SR images of filamentous actin via the direct‐LIVE‐PAINT approach (Figure 1b, c). As with LIVE‐PAINT, we show that direct‐LIVE‐PAINT is dependent on the concentration of the labeled peptide and determines the optimum expression conditions for LifeAct‐14‐EGFP in HEK‐293 cells. We also demonstrate that it is possible to image the dynamic actin structure in live cells, achieving a spatial resolution of 80 nm, with a temporal resolution of 12.5 s. By enabling the tracking of actin dynamics in live cells, we believe that direct‐LIVE‐PAINT with LifeAct‐14‐EGFP will serve as a valuable tool to improve our current understanding of the role played by the cytoskeleton in motility, adhesion, and other cellular processes. Furthermore, direct‐LIVE‐PAINT presents a method for probing a range of biomolecules in SR, provided the peptide has suitable binding kinetics for the target of interest.

## 2 RESULTS

### 2.1 Transfection of LifeAct‐14‐EGFP allows the super‐resolution imaging of actin

**FIGURE 2:**
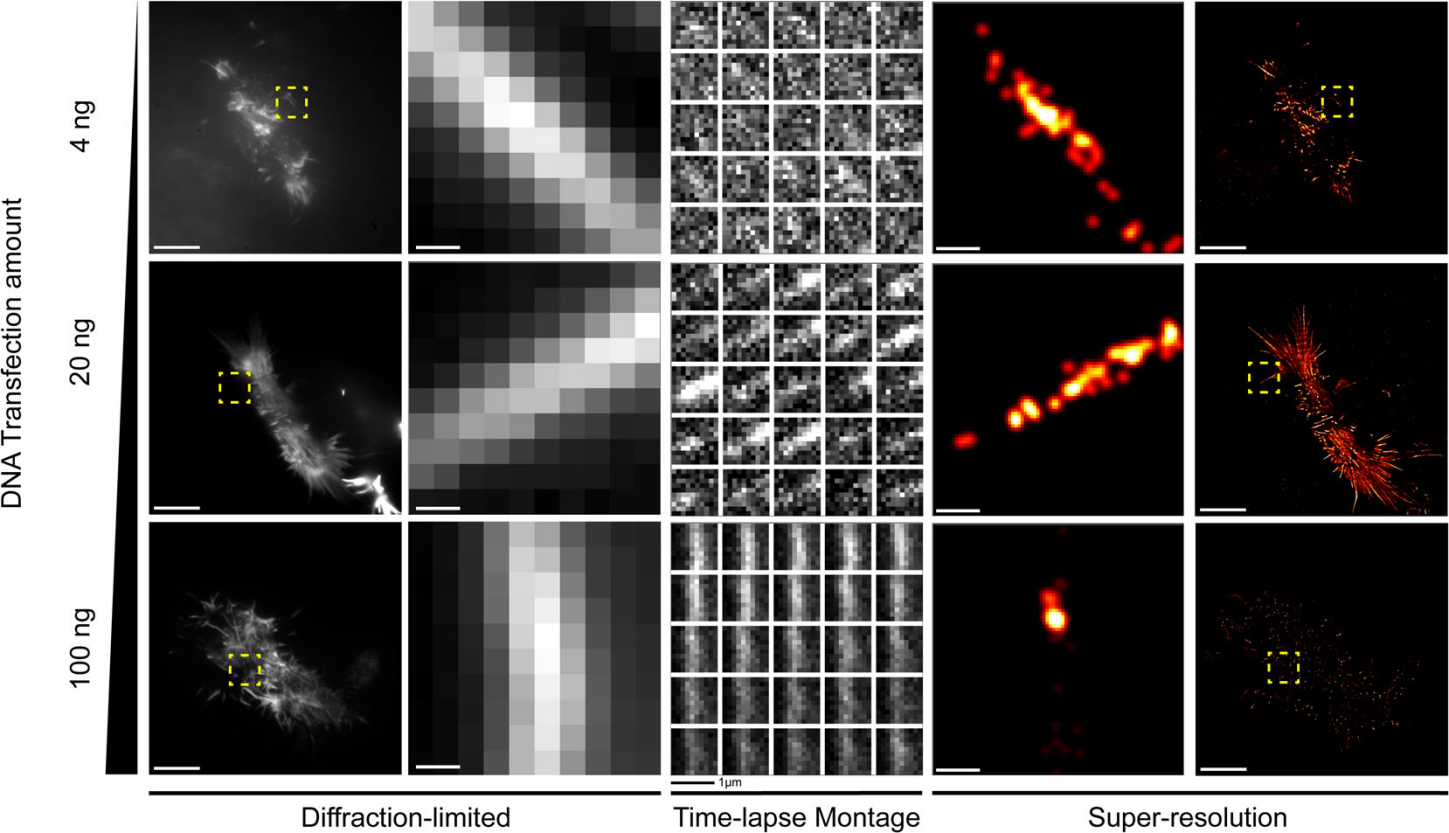
Intensity fluctuations and localizations of LifeAct‐14‐EGFP targeting F‐actin in HEK293 cells in response to a range of DNA transfection amounts. Diffraction‐limited images shown on the left are median intensity Z‐projections where the first column shows an entire field of view (FOV) taken over 2000 frames (100 s, 50 ms exposure, 10 𝜇m scale bar) and the adjacent column shows an ROI from the region in the dashed box shown in yellow over a 300‐frame subset (15 s). The time‐lapse montage column shows intensity fluctuations within the ROI across the 300‐frame subset (12‐frame step [0.6 s], time ‐ left to right and top to bottom). The SR images constructed from the detected localizations in the ROI (0.2 𝜇m scale bar) and then across the entire FOV (10 𝜇m scale bar, 2000 frames) are shown in the far right. Precision threshold <30 nm

As with any SMLM‐based technique, the success of direct‐LIVE‐PAINT relies on having the optimum levels of active fluorophore to enable spatiotemporal separation. We, therefore, investigated how varying the transfection levels of LifeAct‐14‐EGFP affected our ability to distinguish individual fluorophores (Figure 2). At low transfection levels (0.032–0.8 ng DNA), negligible binding was observed. At higher concentrations (4 ng DNA), however, we observed transient binding of LifeAct‐14‐EGFP, allowing the localization of individual emitters (540 localizations/s over 2000 frames). At higher concentrations still (20 ng DNA), the binding rate increased, leading to higher density SR images (1860 localizations/s over 2000 frames, Figure S1) and clear filament structure (spatial resolution of 100 nm as determined by Fourier Ring Correlation [FRC]) (10 Brink, T. RustFRC [Computer software]). At higher expression levels (100 ng DNA) however, individual binding events could no longer be observed due to the saturation of the actin‐binding sites with LifeAct‐14‐EGFP. Based on these observations, a transfection concentration of 20 ng was optimum for LifeAct‐14‐EGFP in live HEK‐293 cells. The images at each transfection amount (Figure 2) are representative examples seen in ~60%–70% of the transfected cell population (data not shown).

### 2.2 Actin filaments in live cells are dynamic structures

Actin is a key component of the cytoskeleton and is responsible for maintaining cell structure and shape (Schnittler et al., 2014). Due to the minimally perturbative nature of direct‐LIVE‐PAINT, and its ability to image in live cells, we used it to track the dynamics of actin filaments.

**FIGURE 3:**
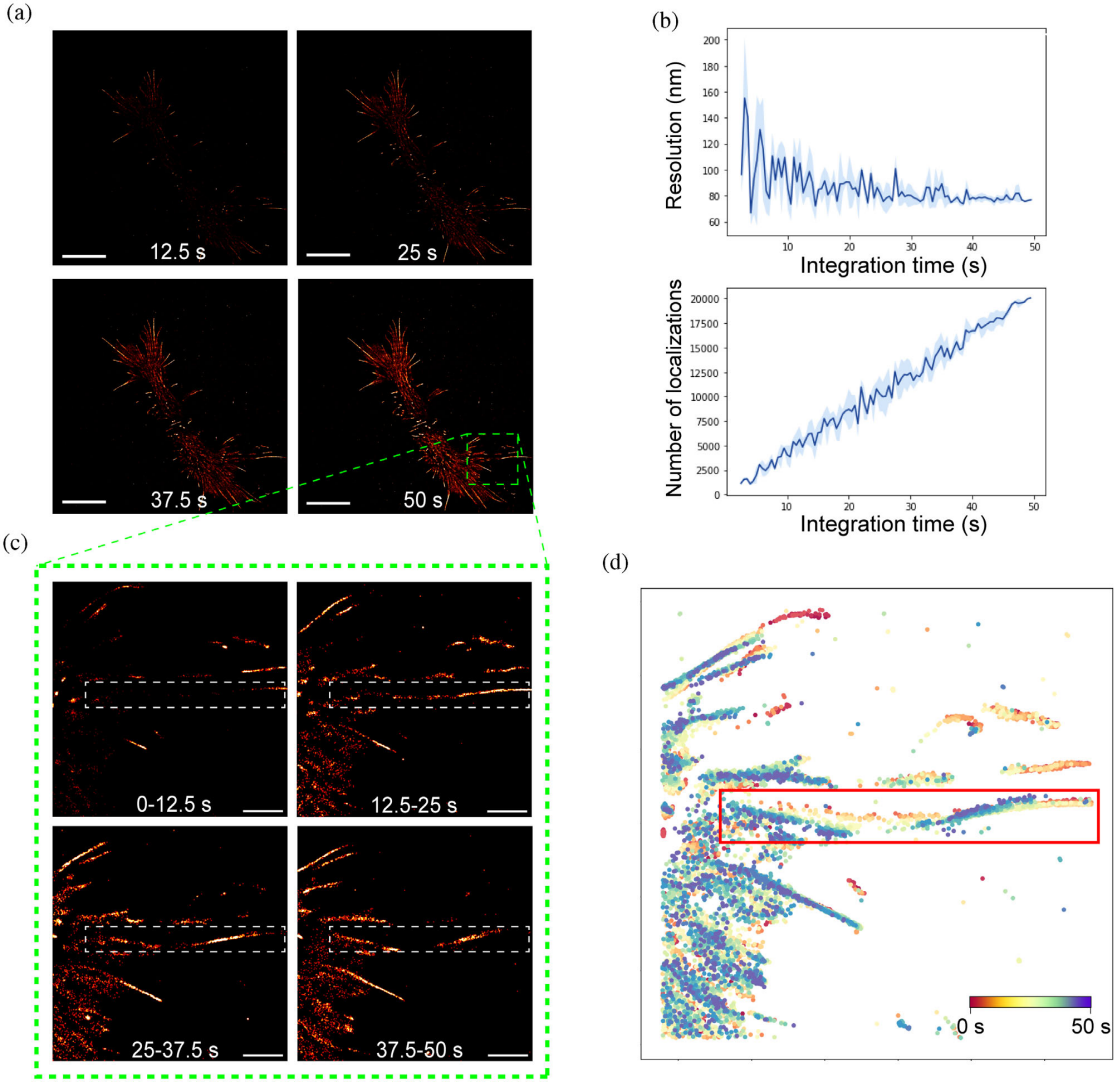
F‐actin structures are dynamic within timescales of imaging and can be detected through direct‐LIVE‐PAINT. (a) SR images of F‐actin imaged with LifeAct‐14‐EGFP at a range of integration times in a HEK293 cell. 10 𝜇m scale bar. (b) Resolution and localization number in response to increasing integration times. Frame subsets were sampled randomly at each integration time for resolution and localization number calculation. Standard deviation error is shown as shaded region (*n* = 3). Precision threshold <20 nm. (c) SR images are shown as a time montage in an ROI (dashed green box from [a]), tracking an actin filament (dashed white box) through the same timeframe. 2 𝜇m scale bar. (d) Map of localizations color‐coded based on time of acquisition across the same timeframe. 2.5 s split, time scales from red to yellow, to green, to blue. Red box indicates the same filament of interest that changes in shape during this timeframe

We first investigated how the imaging time affected the spatial resolution of direct‐LIVE‐PAINT by analyzing subsets of frames generated from LifeAct‐14‐EGFP binding to actin in live HEK‐293 cells (Figure 3). We set a more stringent precision threshold (< 20 nm) to determine whether spatial resolution improves, while still detecting sufficient localizations to discern filament structure (Figure S3). With increasing imaging time (example images in Figure 3a), the spatial resolution increased from ~100 nm to 80 nm (FRC, Figure 3b), and more detail was evident in the images. As localizations were accumulated over time (Figure 3b), a clearer structure was observed. At higher integration times, the resolution appeared to plateau.

To increase the number of localizations further, we applied a less stringent precision threshold (< 30 nm). Although this reduces the spatial resolution (80 nm–100 nm), the increase in the number of localizations (60k vs. 186k) allowed the movement of filaments to be visualized at a time resolution of 12.5 s. Indeed, this can be observed in the time‐montage of the direct‐LIVE‐PAINT images in an ROI (Figure 3c), where the filament (dashed box) clearly changes in shape over 50 s. The SR plot in which the localizations were colored according to the time they were detected (Figure 3d) further illustrates this behavior; the dynamics of the same filament (red box) can be observed throughout the imaging time course.

## 3 DISCUSSION

Several methods now exist to probe protein structures beyond the diffraction‐limit, but many demand sample preparation and imaging conditions that are not compatible with living cells. Some recent methods have allowed live‐cell SR imaging but not without caveats. In the case of stimulated emission depletion microscopy, high excitation laser powers are required and are known to cause cytotoxicity through photodamage (Hell and Wichmann). Structured illumination microscopy, on the other hand, is not as damaging to live samples but demands specialized and expensive equipment (Gustafsson, 2005; Gustafsson et al., 2016). Furthermore, the protein‐of‐interest would need to be fused to a fluorescent tag in both approaches, which can be perturbative to protein function. Finally, SRRF presents another option for live‐cell SR imaging but artifacts have been reported with this approach (Culley et al., 2017, Browne et al., 2019).

In contrast, direct‐LIVE‐PAINT is a minimally perturbative PAINT‐based approach for studying difficult‐to‐tag protein targets at high resolution. We have shown through transient transfection of the 14 AA LifeAct‐14 peptide fused to EGFP that we can observe transient binding events between the probe and F‐actin as single‐molecule localization events, above the background (Figure S2), to construct SR images.

Based on our results presented here, the LifeAct‐14 peptide displays suitable binding kinetics to perform PAINT experiments. Although parameters for *in vivo* binding kinetics were not calculated, the system responded predictably in terms of detecting localizations to varying probe concentrations. The on‐rate responded to the probe concentration by showing a saturated signal at the target and sparse blinking events at 100 ng DNA transfection and insufficient blinking at the 4 ng condition. The off‐rate, however, is key in determining the suitability of a probe for PAINT, and a *k*_off_ ~ 1 s^−1^ is suitable for LIVE‐PAINT studies (Oi et al., 2020a).

We were also able to study actin dynamics with direct‐LIVE‐PAINT. The spatial resolution of images generated using SMLM is dependent on both the precision of each emitter localized and the density of the localizations. To achieve the highest resolution, it can therefore be necessary to image over several minutes, resulting in the loss of temporal resolution. Thus, there is a compromise to be made between achieving the highest spatial and temporal resolution. By making the precision threshold less stringent we were able to track dynamic structures at a temporal resolution of 12.5 s, suggesting the technique's wider applicability in studying live‐cell processes.

## 4 CONCLUSION

In summary, we demonstrate that direct‐LIVE‐PAINT can be used to probe protein targets in live cells. We used direct‐LIVE‐PAINT with LifeAct14‐EGFP to characterize the structure and dynamics of F‐actin at the nanoscale. This is the first LIVE‐PAINT‐based imaging in mammalian cells. Although shown with an actin‐binding peptide here, other classes of peptides, such as nanobodies (Muyldermans et al., 2009), affibodies (Nord et al., 1997), and DARPins (Binz et al., 2003) that target various proteins in mammalian cells could potentially be used with direct‐LIVE‐PAINT.

## 5 MATERIALS AND METHODS

### 5.1 DNA amplification and purification

LifeAct‐14‐EGFP plasmid was received as agar stabs (#158750, Addgene) (Kumari et al., 2020). Cultures were grown up according to standard protocols. Plasmid DNA was purified using a QIAGEN Spin Miniprep Kit according to manufacturer protocols and concentration and purity were assessed on a NanoDrop™ Microvolume spectrophotometer. Extracted DNA was stored at −20°C until ready for transfection.

### 5.2 Mammalian cell culture and transfection

HEK293 cells were cultured according to standard protocols (ATCC). Briefly, cells were cultured in DMEM supplemented with 10% FBS, 1% L‐glutamine, and 1% Pen/Strep in T25 flasks. Once ~70% confluent, cells were detached with TrypLE (Gibco) by adding 0.5 ml and incubating for 2–3 mins in the incubator at 37°C. Then, 4 ml of low‐fluorescence DMEM, without phenol Red (Gibco™, Cat no. 21063–029) was added and the cell density of the suspension was counted with an automated cell counter (Countess II, Thermofisher Scientific) using 0.4% Trypan Blue at a 1:1 cell:dye ratio (1:2 dilution) Ibidi imaging plates (#81817) were seeded at 15 k cells in 100 𝜇l per well (1.5e5 cells/ml) and allowed to adhere for ~20 min at room temperature.

Meanwhile, transfection reagents (Lipofectamine™ 3000 Transfection Reagent, Invitrogen™) were made up according to the manufacturer's protocol. Briefly, the P3000 reagent was diluted into Opti‐MEM™ (Gibco™) and mixed with different amounts of DNA based on the transfection condition. Next, Lipofectamine 3000 Reagent diluted in Opti‐MEM was added and mixed thoroughly by pipeting up and down. The lipofectamine‐DNA mixture was incubated for 10 min at room temperature before being added to the cells at 25 𝜇l per well. Plates were incubated for 24 h at 37° C, under 5% CO_2_, prior to imaging.

Because this is a proof of principle experiment, we were satisfied with empirically optimizing the DNA amount used in transfection to identify the amount that gave appropriate binding behavior to allow SMLM.

### 5.3 TIRF microscopy

All imaging was performed based on a previously published protocol (Oi et al., 2020b). Briefly, a home‐built TIRF instrument using an inverted Nikon® TI2 microscope was used with a heated stage incubator to maintain humidity and temperature at 37°C. EGFP was excited with laser light at a wavelength of 488 nm and the emission was collected by the same objective by separating it from the returning TIR beam with the use of a dichroic mirror. Images were acquired at an exposure of 50 ms and a laser power density of 3.5 W/cm^2^. Higher power densities are likely to cause toxicity in live cells. Fewer localizations were expected from initial frames as the sample bleached down to a suitable labeling density. ImageJ was used for all data acquisition and analysis along with the Micromanager software for microscope automation.

All SR image construction was performed using the FIJI (ImageJ2; Version 2.3.0) plug‐in ThunderSTORM (version dev‐2016‐09‐10‐b1) by running the *Run analysis* command (Ovesný et al., 2014). Localizations were visualized using the *Normalized Gaussian* method (width = precision) along with a < 30 nm precision threshold unless otherwise stated.

Resolution and localization number calculations were performed using the RustFRC python package (10 Brink, T. RustFRC [Computer software]) and the script is available at https://doi.org/10.5281/zenodo.7290477. Briefly, consecutive frame subsets of a specific size, determined by the integration time, were randomly sampled from the total frames dataset for resolution and localization number calculation.

## AUTHOR CONTRIBUTIONS

**Dirk‐Jan Kleinjan:** Methodology (supporting); resources (supporting); writing – review and editing (supporting). **Curran Oi:** Conceptualization (supporting); investigation (supporting); writing – review and editing (supporting). **Zoe Gidden:** Investigation (supporting); writing – review and editing (supporting). **Susan J. Rosser:** Methodology (supporting); resources (supporting); writing – review and editing (supporting).

## CONFLICT OF INTEREST

The authors declare that the research was conducted in the absence of any commercial or financial relationships that could be construed as a potential conflict of interest.

## Supporting information


**Appendix S1.** Supporting Figures
