# Supplementary material for: Live‐cell super‐resolution imaging of actin using LifeAct‐14 with a PAINT‐based approach
Source: Protein Sci. 2023 Feb 1;32(2):e4558. doi: 10.1002/pro.4558 (PMC9878614; doi:10.1002/pro.4558)
Supplement: Supplementary file 1 — Appendix S1. Supporting Figures [file PRO-32-e4558-s001.pdf]

Supplementary Figures

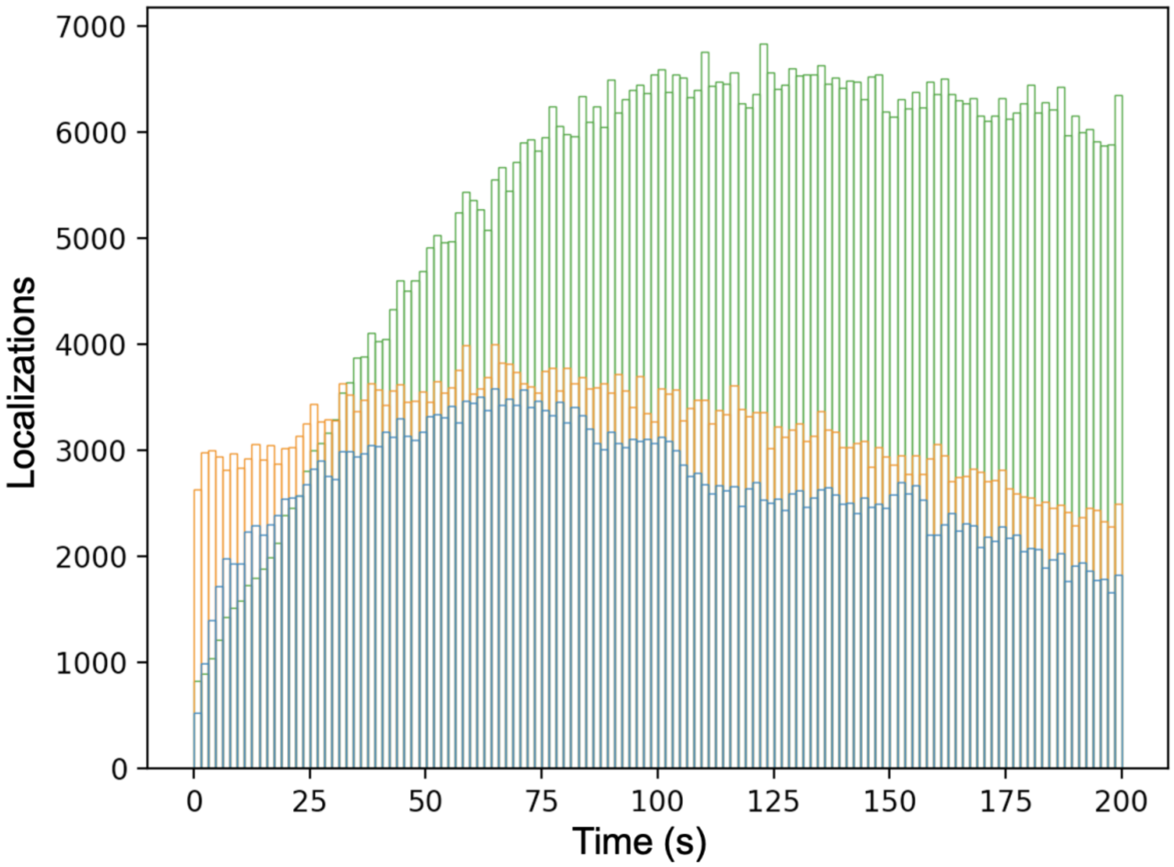

Supplementary Figure S1: Plot of localizations against time from HEK293 cells (n=3) labelled with LifeAct-14-EGFP at 20 ng. Each color represents a different FOV from the same experiment. 50 ms exposure, 4000 frames acquisition. Precision threshold < 30 nm.

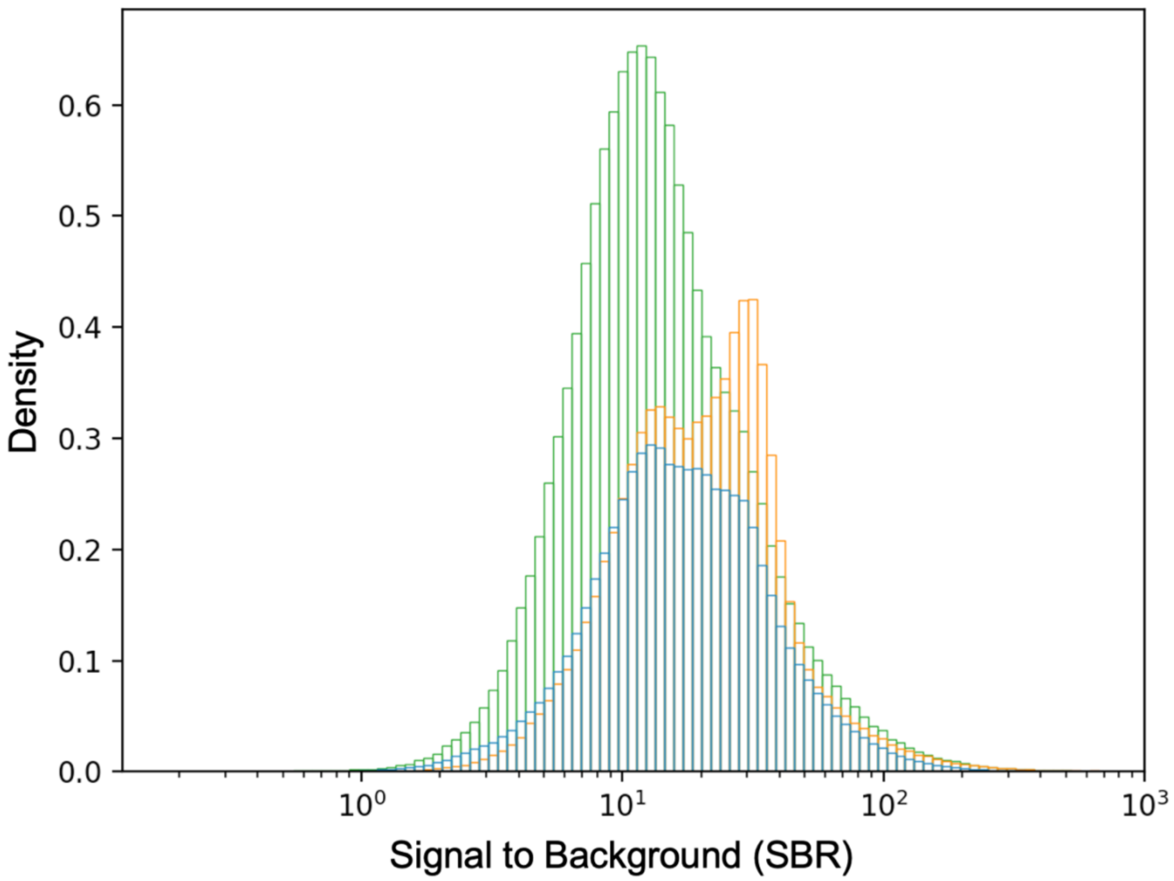

Supplementary Figure S2: Distribution of signal to background of localizations in HEK293 cells (n=3) labelled with LifeAct-14-EGFP at 20 ng. Each color represents a different FOV from the same experiment. 50 ms exposure, 4000 frames acquisition. Precision threshold < 30 nm.

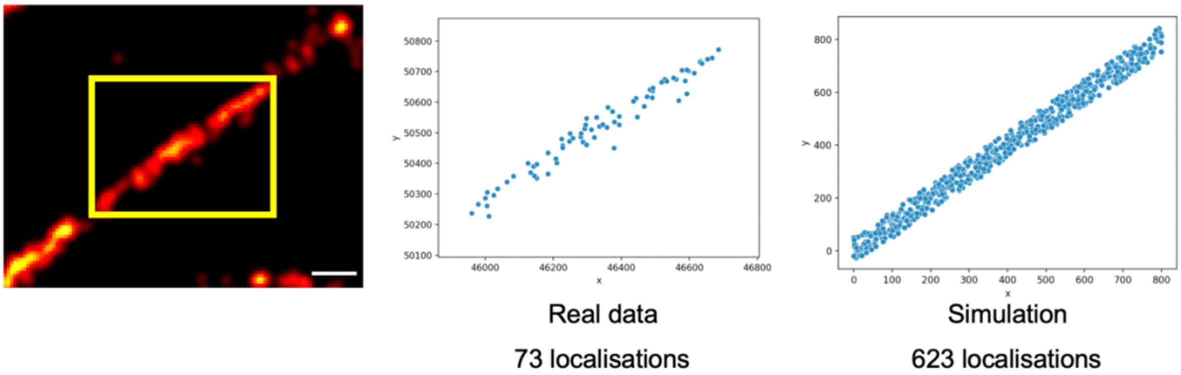

Supplementary Figure S3: Comparison of localization density on a filament ROI compared to simulated data at the Nyqvist sampling limit. Panel on the left shows ROI (yellow box) in a super-resolution image of F-actin labelled with LifeAct-14-EGFP. 0.2 μm scale bar. Center panel shows localizations within the ROI as a scatter plot. Panel on the right shows simulated localization pattern in a region of similar size if the density was at the Nyqvist sampling limit. Filament was estimated to be a linear 70 nm fiber with a diameter of 6 nm. Localization position was randomly determined as a normal distribution around the filament within ± 50 nm.
